# Supplementary material for: Cognitive function in non-hospitalized patients 8–13 months after acute COVID-19 infection: A cohort study in Norway
Source: PLoS One. 2022 Aug 22;17(8):e0273352. doi: 10.1371/journal.pone.0273352 (PMC9394790; doi:10.1371/journal.pone.0273352)
Supplement: S1 Table — Comparison between those completing CANTAB assessment during the follow-up visit (n = 233) and non-participants/not responding to CANTAB (n = 225). (PDF) [file pone.0273352.s001.pdf]

S1 Table. Non-response analysis among those that responded to the first survey (n=458). Comparison between those completing CANTAB assessment during the follow-up visit (n=233) and non-participants/not responding to CANTAB (n=225)

	Completing CANTAB			Not completing CANTAB or non-participation			*P*
	N	n	Mean (SD) /Proportion	N	n	Mean (SD) /Proportion	
Age	233		49.8 (14.7)	225		49.1 (16.0)	0.62
Sex, males	233	95	0.41		107	0.48	0.144
Highest attained education	233						0.47
Primary school (<10 years)		17	0.07		24	0.11	
Secondary school (10–12 years)		95	0.41		79	0.35	
University level, < 4 years		60	0.26		61	0.27	
University level, ≥ 4 years		61	0.26		61	0.27	
Marital status	233						0.65
Single/separated/divorced		56	0.24		46	0.21	
Married/cohabiting		168	0.72		168	0.75	
Widowed		9	0.04		10	0.04	
Born in Norway	231	200	0.87	223	182	0.82	0.148
No. of symptoms during acute COVID-19	233			225			0.026
0–5		52	0.22		54	0.24	
6–9		78	0.33		98	0.44	
10–23		103	0.44		73	0.32	
EQ-5D Index	232		0.792 (0.011)	224		0.840 (0.011)	0.002
