# Supplementary material for: Cognitive function in non-hospitalized patients 8–13 months after acute COVID-19 infection: A cohort study in Norway
Source: PLoS One. 2022 Aug 22;17(8):e0273352. doi: 10.1371/journal.pone.0273352 (PMC9394790; doi:10.1371/journal.pone.0273352)
Supplement: S2 Table — (PDF) [file pone.0273352.s002.pdf]

S2 Table. Cognitive test scores for subjects ≥60 years and comparison with norm population.

			Raw score			Z-score*			P**	Z-score <-1.5	
Test	Description	Score (range)	n	Mean	SD	n	Mean	SD		No.	%
Delayed matching to sample (DMS)	DMS Percent Correct (All trials containing a delay)	0 to 100 (best)	56	76.8	10.8	55	-0.68	0.94	<0.001	7	12.7
One Touch Stockings of Cambridge (OTS), standard version	No. of OTS Problems Solved on First Choice	0 to 15 (best)	56	8.6	3.5	55	-0.38	1.14	0.030	13	23.6
Rapid visual information processing (RVP), 3 targets	RVP A' (A prime) measures a subject's sensitivity to the target sequence (string of three numbers), regardless of response tendency.	0 to 1 (best)	52	0.87	0.05	51	-0.57	0.61	<0.001	4	7.8
Spatial working memory (SWM), recommended standard 2.0 extended	SWM Between Errors. No. of times the subject incorrectly revisits a box in which a token has previously been found. Across all assessed trials.	0 to 153 (worst)	56	16.1	9.5	55	-0.49	0.96	<0.001	4	7.2

* higher z-score is better performance

**Z-score comparison with norm population (mean=0)
