# Supplementary material for: Cognitive function in non-hospitalized patients 8–13 months after acute COVID-19 infection: A cohort study in Norway
Source: PLoS One. 2022 Aug 22;17(8):e0273352. doi: 10.1371/journal.pone.0273352 (PMC9394790; doi:10.1371/journal.pone.0273352)
Supplement: Attachment — Submitted filename: Response_to_reviewers_13jun2022_KSCL.docx [file pone.0273352.s003.docx]

**Comments to editor’s and reviewers’ comments**

When submitting your revision, we need you to address these additional requirements.

1. Please ensure that your manuscript meets PLOS ONE's style requirements, including those for file naming. The PLOS ONE style templates can be found at <https://journals.plos.org/plosone/s/file?id=wjVg/PLOSOne_formatting_sample_main_body.pdf> and <https://journals.plos.org/plosone/s/file?id=ba62/PLOSOne_formatting_sample_title_authors_affiliations.pdf>

***Done***

2. Please amend your current ethics statement to address the following concerns:

a) Did participants provide their written or verbal informed consent to participate in this study?

b) If consent was verbal, please explain i) why written consent was not obtained, ii) how you documented participant consent, and iii) whether the ethics committees/IRB approved this consent procedure.

***Done (lines 194-196)***

3. Please include your full ethics statement in the ‘Methods’ section of your manuscript file. In your statement, please include the full name of the IRB or ethics committee who approved or waived your study, as well as whether or not you obtained informed written or verbal consent. If consent was waived for your study, please include this information in your statement as well.

***Done***

**Reviewers' comments:**

Reviewer's Responses to Questions

Comments to the Author

1. Is the manuscript technically sound, and do the data support the conclusions?

The manuscript must describe a technically sound piece of scientific research with data that supports the conclusions. Experiments must have been conducted rigorously, with appropriate controls, replication, and sample sizes. The conclusions must be drawn appropriately based on the data presented.

Reviewer #1: Yes

Reviewer #2: Yes

________________________________

2. Has the statistical analysis been performed appropriately and rigorously?

Reviewer #1: Yes

Reviewer #2: Yes

________________________________

3. Have the authors made all data underlying the findings in their manuscript fully available?

The PLOS Data policy<<http://www.plosone.org/static/policies.action#sharing>> requires authors to make all data underlying the findings described in their manuscript fully available without restriction, with rare exception (please refer to the Data Availability Statement in the manuscript PDF file). The data should be provided as part of the manuscript or its supporting information, or deposited to a public repository. For example, in addition to summary statistics, the data points behind means, medians and variance measures should be available. If there are restrictions on publicly sharing data—e.g. participant privacy or use of data from a third party—those must be specified.

Reviewer #1: No

Reviewer #2: Yes

***A data availability statement was specified in the original submission.***

4. Is the manuscript presented in an intelligible fashion and written in standard English?

PLOS ONE does not copyedit accepted manuscripts, so the language in submitted articles must be clear, correct, and unambiguous. Any typographical or grammatical errors should be corrected at revision, so please note any specific errors here.

Reviewer #1: Yes

Reviewer #2: Yes

Reviewer #1: The authors investigated the cognitive function for non-hospitalized patients with PCR-confirmed COVID-19 patients. They longitudinally followed them.

At median 11 (range 8–13) months after PCR positivity, cognitive scores for short term memory, visuospatial processing, learning and attention were lower than norms (p≤0.001).

At median 11 months after out-of-hospital SARS-Cov-2 infection, minor cognitive impairment was seen with little association between COVID-19 symptom severity and outcome.

I will raise some concerns before publication.

Major

#1

Why did not the authors perform cognitive testing for dyspnea group? The authors hypothesized that the cognitive sequelae were associated with hypoxia. Then, dyspnea group should be conducted cognitive testing.

Could you elaborate on “Because of risk of attrition”?

***As stated in the section “Follow-up survey and visit” the follow-up program was differentiated to allow for a slightly different layout adapted to the different groups. This was as the examinations performed were lengthy and risked leading to reduced willingness to participate in the follow-up – i.e. higher levels of attrition.***

***The examination program was stratified according to findings from the 3-month survey. As fatigue has been reported as a major subjective complaint in late COVID-19, we wanted to ensure that we included patients with this complaint in the cognitive assessment.***

***Not including those with dyspnea only at 3 months, as well as not including all asymptomatic patients, were decisions made based on logistic reasons and capacity, while including all patients with fatigue at 3 months.***

***While this was, in hindsight, perhaps unfortunate, we nevertheless performed the additional sensitivity analyses described as an attempt to control whether these procedures biased our results. As stated, the sensitivity analyses performed did not affect the main outcomes.***

#2

As the authors pointed out, the repone rate was low. Selection bias should be considered.

Are there any differences between response and non-response to questionnaire of survey 1?

If any, discuss it. And are there any differences between response and non-response to CANTAB of survey 2? If any, discuss it.

***We had limited information on non-respondents in Survey 1, but an analysis of this has previously been presented. The discussion on this has been revised/expanded (lines 350-355):***

*The respondents in the first round were older (mean age on 1 June 2020 49.5 (SD 15.3) vs. 43.9 (17.3) years for non-respondents (p < 0.001) and comprised a larger proportion of women (256 (56%) vs. 219 (46%), p = 0.005 (1). The response rates in seven aggregated geographical districts within the catchment areas of the hospitals ranged from 26% to 65%, with lowest rates in three districts of Oslo that have a large proportion of immigrants in the population (2). This may have introduced bias in the sample.*

(1)	Stavem K, Ghanima W, Olsen MK, Gilboe HM, Einvik G. Prevalence and Determinants of Fatigue after COVID-19 in Non-Hospitalized Subjects: A Population-Based Study. Int J Environ Res Public Health. 2021;18(4). Epub 2021/03/07. doi: 10.3390/ijerph18042030. PubMed PMID: 33669714; PubMed Central PMCID: PMCPMC7921928.)

(2) Stavem K, Ghanima W, Olsen MK, Gilboe HM, Einvik G. Persistent symptoms 1.5-6 months after COVID-19 in non-hospitalised subjects: a population-based cohort study. Thorax. 2021;76(4):405-7. Epub 2020/12/05. doi: 10.1136/thoraxjnl-2020-216377. PubMed PMID: 33273028; PubMed Central PMCID: PMCPMC7716295.)

***We compared respondents completing cognitive testing with CANTAB (n=233) with non-participants/participants not having cognitive assessment (n=225). This has been added as a supplementary table (S1 Table) and presented briefly in Results (lines 215-218), and a comment has been added in Discussion (lines 358-360).***

Minor

#3

In Result, Sample

“191 of these patients refused further follow-up” should be “19 of these patients refused further follow-up”.

***Corrected.***

#4

Please mention the COVID-19 infectious situation, such as local infections prevalence and mutation of prevalent SARS-CoV-2.

***In June 2020, at the time of study recruitment, there was no routine reporting of virus variants. A summary report based on a limited sample (n=91) in July 2020 reported that the first cases were B, and the majority during the first few months of 2020 was subtype B.1, and less commonly B.1.1 and B.1.1.1.***

***Some sentences about this have been added to the Methods (lines 76-80):***

*The study participants were recruited during the first wave of COVID-19 in Norway until 1 June 2020, when a total of 8,443 laboratory-confirmed cases had been reported, or 157 cases per 100,000 inhabitants, and 1,068 patients with COVID-19 had been hospitalized (3). Based on a limited national sample, the dominating subtype during this period was B.1, and less commonly B.1.1 and B.1.1.1 (4).*

3. Folkehelseinstituttet. COVID-19 Ukerapport uke 22, 3. Jun 2000 [Available from: <https://www.fhi.no/contentassets/8a971e7b0a3c4a06bdbf381ab52e6157/vedlegg/forste-halvar--2020/2020.06.03-ukerapport-uke-22-covid-19.pdf>
4. Folkehelseinstituttet. COVID-19 Ukerapport uke 27, 8. Jul 2000

<https://www.fhi.no/contentassets/8a971e7b0a3c4a06bdbf381ab52e6157/vedlegg/andre-halvar--2020/2020.07.08-ukerapport-uke-27-covid-19-versjon-2.pdf>

Over all, the paragraph division is bad and it is difficult to read.

***This has been checked, however referee #2 has a contrasting view “the manuscript is well written, especially Discussion”. Further down he/she states “the introduction is well written”. Some changes representing a compromise between these two views have been made in the introduction section (lines 42-51)***

Reviewer #2: Overall statement

This study aims to evaluate the effect of Covid-19 infection on cognitive function in non-hospitalized patients. The researchers found that late after a Covid-19 infection, non-hospitalized patients showed only slightly lower cognitive test scores. The research question is clearly outlined. The authors used a detailed cognitive test battery. The results seem to be valid and reliable. In my opinion the sample size is limited for a population based study.

Strengths and impact

This is a longitudinal study that includes evaluations with a good quality cognitive test battery (CANTAB). This battery evaluates many cognitive domains, especially executive functions. The title of the study is informative. The study aim is interesting since they measured the cognitive effects after a Covid-19 infection in a non-hospitalized group with low comorbidity scores. The references at the end of the text are up to date. The current knowledge about the topic are given in detail. Statistical methodology seems to be nice (using Z scores). The manuscript is generally well written (especially discussion).

***Thank you!***

Minor weaknesses

1. Even if they designed a longitudinal study, the reserchers very found mild differences in cognitive scores (%4-14). Since this is a cognitive study. The results must be discussed further in relation to participant age, educational status and comorbid conditions. It may be possible to find significant cognitive differences in an elderly population with a larger sample size.

***Yes, we agree that the differences are mild in cognitive scores when comparing a normal population with our data on previously COVID-19 afflicted individuals. Exactly this was our stated objective. Our intention was thus not to specifically focus on an elderly population. However, it is clear that in any study of objectively tested cognitive function, co-variates must include among others age, education and comorbidity. We had therefore, a priori, decided to include these co-variates as possible confounders. However, we cannot from this exclude that significant differences could have been found in a larger population of elderly. Therefore, as the referee suggested, we have “post hoc” made a supplementary analysis of the elderly population in our sample (S2 Table) See also response to comment # 5.***

2. The introduction is well written. The author wrote that they aimed to determine the prevalence of cognitive deficits 8-13 months after Covid-19 infection. The author must explain the importance/meaning of this time interval (8-13 months) since they wrote it in the study aim and the title.

***Our intention was to study late cognitive function after an out-of-hospital COVID-19 infection, as stated in the final part of the introduction as most studies hitherto have only focused on shorter time windows. Around one year (with plus and minus depending on logistic reasons) was chosen as a compromise between a time period when most patients would be expected to have recovered from acute symptoms and a reasonably attainable long-term follow-up period. The interval of 8-13 months reflects the actual time of the assessments in the study.***

3. Did any of the patients have CT documented pulmonary Covid disease? The Figure legends are missing.

***This was not the focus of our study, and we do not have CT documentation of the acute COVID-19 phase as these patients were not admitted to hospital.***

***Figure captions have now been inserted in the running text (Fig 1 and Fig 2), instead of at the end of the manuscript.***

4. The researcher divided the patients with a Covid-19 history into 3 groups according to their symptoms (fatigue, dyspnea & fatigue, no dyspnea or fatigue). I think that this classification makes the study a little bit confusing. If I were to do a patient classification for non-hospitalized Covid-19 patients I would divide them into 2 groups such as; asymptomatic/mild symptomatic and symptomatic (including dyspnea and apparent fever). Maybe if they made 2 patient groups with a different methodology, they could have included dyspnea only patients and the remaining asymptomatic patients to increase the sample size.

***Rather than making an arbitrary categorical division as suggested here we (again a priori) chose to count symptoms based on an extensive symptoms reporting list as well as specifically focussing on symptoms that may suggest cerebral involvement.***

***As fatigue has been reported as a major subjective complaint in late COVID-19, we wanted to ensure that we included patients with this complaint in the cognitive assessment.***

***Not including dyspnea only patients, as well as not including all asymptomatic patients, were decisions made based on logistic reasons and capacity, while including all patients with fatigue at 3 months (see also comment to reviewer #1, item #1)***

5. Table 1. is informative. Table 3. is easy to understand and definitive. The regression analysis did not reveal any significance except patient age (Table 5.). The author may want to make a subgroup analysis in older participants to see which cognitive domains were effected after an acute Covid-19 infection.

***We have made a supplementary analysis (S2 Table), corresponding to Table 3, in subjects above 60 years of age. This was similar to the findings in the total sample, but with more problems on the OTS, i.e. in executive function, spatial planning and working memory.***

***Even though this analysis was not pre-planned and therefore liable to possible spurious findings, we think it is interesting and worthy of a comment, which has now been added.***

***We thank the referee for the suggestion!***

6. In the results/sample section the number of patients participated in CANTAB was given as 233. In Table 2. the number seems to be 234?

***This has been corrected to reflect n=233 also in Table 2.***

7. Even if the author made adjustments for possible confounders, there may be other confounders not taken into account. There is no control group as the author mentioned. Therefore, it would be better if the author writes some more possible limitations.

***The section in the Discussion on possible limitations has been expanded (lines 349-360).***

Major weaknesses

1. In my opinion the sample size is limited for a population based study.

***We have also commented on this point in the limitations section of the Discussion.***
