# Supplementary material for: Cognitive function in non-hospitalized patients 8–13 months after acute COVID-19 infection: A cohort study in Norway
Source: PLoS One. 2022 Aug 22;17(8):e0273352. doi: 10.1371/journal.pone.0273352 (PMC9394790; doi:10.1371/journal.pone.0273352)
Supplement: Attachment — Submitted filename: Rebuttal_letter_2.docx [file pone.0273352.s004.docx]

**Rebuttal letter.**

Comments to reviewer #1:

Reviewer #1: The manuscripts were well revised.
All my concerne were satisfied.

I raise one addition comments.
Minor
The title should be revised,
Cognitive function in non-hospitalized patients 8–13 months after acute COVID-19
infection: a cohort study
===>
Cognitive function in non-hospitalized patients 8–13 months after acute COVID-19
infection: a cohort study in Norway.

**Response: The title has been revised, as suggested**
